# Beyond “Living Well”: Toward Socially Just Cultural Narratives About Living With Dementia

**DOI:** 10.1093/geroni/igaf122.970

**Published:** 2025-12-31

**Authors:** Janelle Taylor, Nancy Berlinger

**Affiliations:** University of Toronto, Toronto, Ontario, Canada; The Hastings Center, Garrison, New York, United States

## Abstract

This paper considers how cultural narratives function when an aging society is reflecting on itself and what it wants for fellow members of this society, specifically, people facing dementia and people who are dementia caregivers. It explores the moral imagination required to develop socially just cultural narratives for aging societies that will endure under non-ideal conditions. Moral imagination is the capacity to think about current and future challenges in ways that aim at better lives and greater justice. Socially just cultural narratives are the stories embedded in these tasks that convey ideas and values about how to support better lives for disadvantaged members of a society, alongside other important goals such as effectiveness and sustainability. When used and acted upon by those with the power to drive change, these narratives should help to produce opportunities for better lives and greater justice under real-world conditions. The author, an anthropologist, studies the social, cultural, and political dimensions of illness and medicine in North America, with special attention to dementia and care.

